# Supplementary material for: Single-cell atavism reveals an ancient mechanism of cell type diversification in a sea anemone
Source: Nat Commun. 2023 Feb 16;14:885. doi: 10.1038/s41467-023-36615-9 (PMC9935875; doi:10.1038/s41467-023-36615-9)
Supplement: Supplementary file 1 — Supplementary Information [file 41467_2023_36615_MOESM1_ESM.pdf]

Supplementary Information for:

Single-cell atavism reveals an ancient mechanism of cell type diversification in a sea anemone

Leslie S. Babonis [1,2], Camille Enjolras[1], Abigail J. Reft[3,4], Brent M. Foster[1], Fredrik Hugosson[1], Joseph F. Ryan[1,5], Marymegan Daly[6], and Mark Q. Martindale[1,5]

Correspondence to: lsb257@cornell.edu

This PDF file includes:

Supplementary Figures 1 to 5

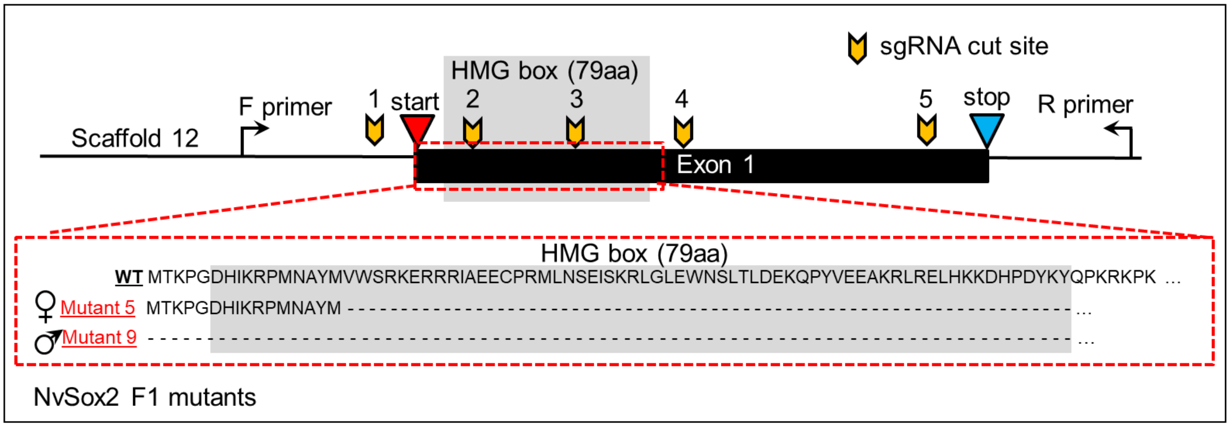

Supplementary Fig. 1: CRISPR/Cas9-mediated genome editing for knockout of *NvSox2* in *N. vectensis*. *NvSox2* is a single-exon gene with a conserved DNA binding domain (HMG box). Targets of guide RNAs (sgRNA cut site 1-5) and genotyping primers (F primer, R primer) are indicated. Mutations sequenced from two F1 adult polyps (Mutant 5, Mutant 9) are indicated.

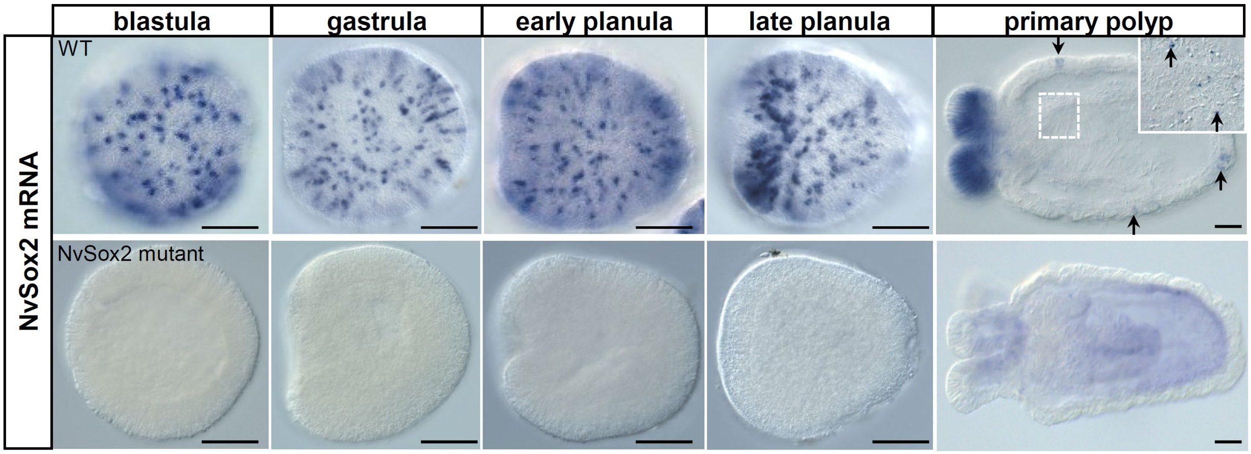

Supplementary Fig. 2: Knockout of *NvSox2* results in loss of *NvSox2* expression throughout embryogenesis in *N. vectensis*. Expression of *NvSox2* is abolished in *NvSox2* mutants relative to wild type animals (WT). Insets show surface detail of boxed region; arrows indicate individual cells expressing *NvSox2*. Each panel is representative of the expression pattern recovered from at least 10 individuals at each stage. Oral pole is to the left in all images. All scale bars: 50um.

.

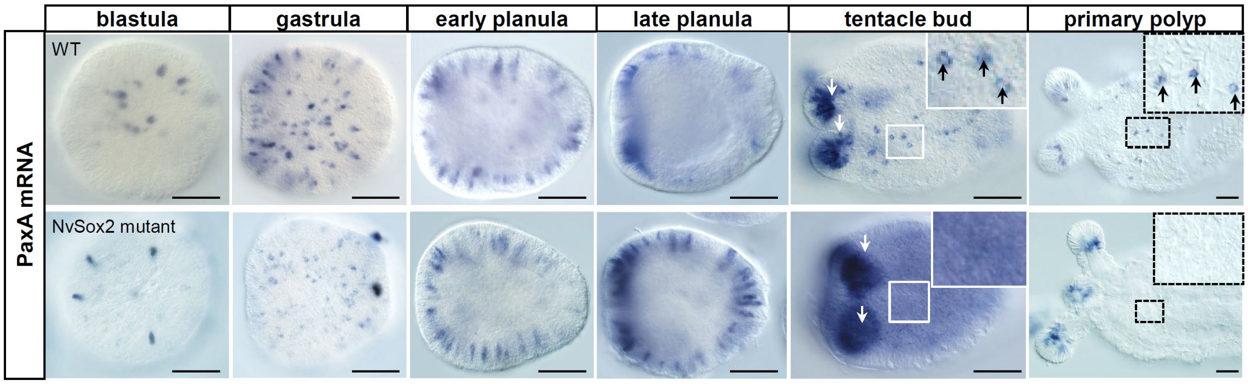

Supplementary Fig. 3: Knockout of *NvSox2* results in loss of *PaxA* expression in the body wall of *N. vectensis*. Expression of *PaxA* is abolished in the body wall of *NvSox2* mutants relative to wild type animals (WT). Insets show surface detail of boxed regions; arrows indicate individual cells expressing *PaxA*. Each panel is representative of the expression pattern recovered from at least 10 individuals at each stage. Oral pole is to the left in all images. All scale bars: 50um.

.

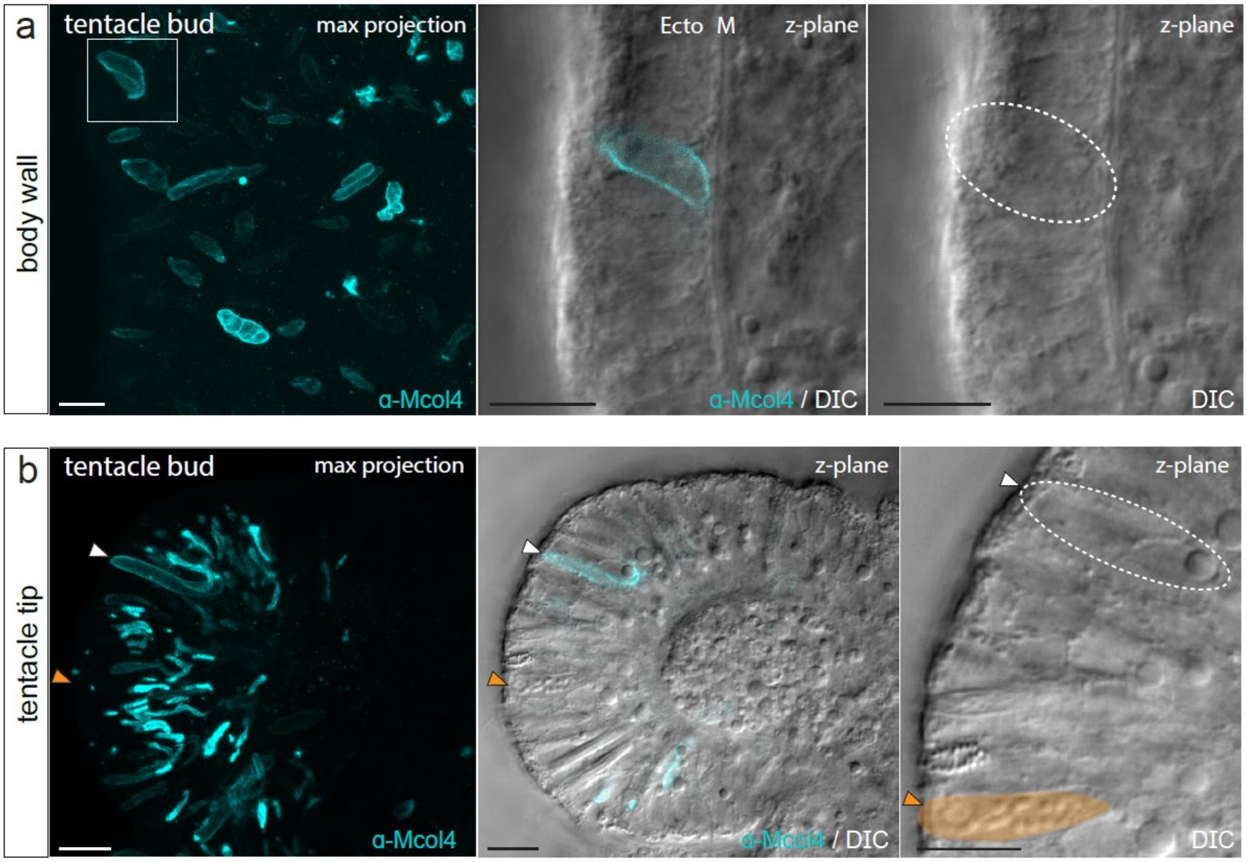

Supplementary Fig. 4: Detection of immature stinging cells with α-Mcol4 antibody in *NvSox2* mutant animals. a α-Mcol4 labeled mutant stinging cells in the body wall shown in a 3D rendered z-stack (max projection) and an individual optical section (z-plane) (N = 4 animals). Cells labeled with α-Mcol4 are immature and do not yet have a capsule that is visible in DIC (white circle). Ecto – ectoderm, M – mesoglea. **b** α-Mcol4 labels immature stinging cells in the tentacle tip (N = 4 animals). A developing large piercing cell (basitrichous isorhiza nematocyte) is labeled with α-Mcol4 (white arrowhead); at this stage the capsule is not yet visible with DIC (white circle). A mature mutant cell (orange arrowhead) has a capsule that can clearly be seen in DIC (false colored orange) but is not labeled with α-Mcol4 antibody. All scale bars: 10um.

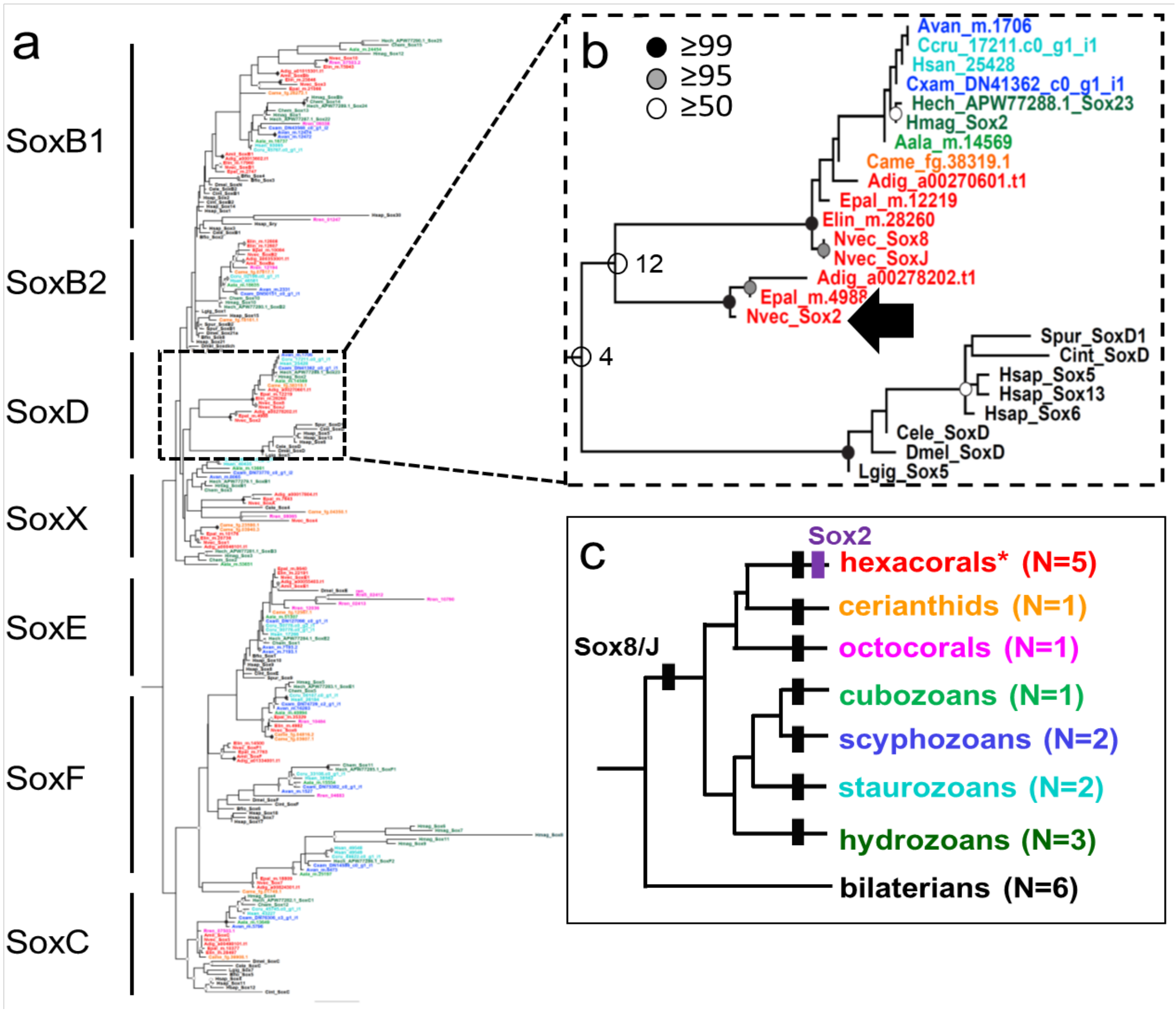

Supplementary Fig. 5: Maximum likelihood phylogeny of *Sox* genes translated from 15 cnidarian and 6 bilaterian transcriptomes. a Complete *Sox* tree showing recovery of clades B1, B2, C, D, E, and F in this analysis. A clade of *Sox* genes restricted to cnidarians (represented by Nvec_SoxA, Nvec_Sox1, and Nvec_Sox4) was also recovered and is labeled SoxX. See Supplementary Data 1 for alignment. **b** Detail of clade indicated by dotted lines including bilaterian *SoxD* genes and the clade including *NvSox2* (Nvec_Sox2) and *NvSox8/J* (Nvec_Sox8/J). There is low support for the sister relationships of these clades (open circles). Support from 500 bootstraps is indicated by black, grey, and white circles at nodes. Where open circles are used, the bootstrap value is provided. **c** Taxonomic sampling scheme and hypothesized origin of *NvSox2* and *NvSox8/J* orthologs. N = number of species from each group included in analysis; colors in a,b indicate taxonomic affiliation as shown in c. See Supplementary Methods for

taxonomic abbreviations in a,b. *Non-cerianthid hexacorals (this group currently lacks an accepted name).
