# Supplementary material for: Single-cell atavism reveals an ancient mechanism of cell type diversification in a sea anemone
Source: Nat Commun. 2023 Feb 16;14:885. doi: 10.1038/s41467-023-36615-9 (PMC9935875; doi:10.1038/s41467-023-36615-9)
Supplement: Supplementary file 3 — Description of Additional Supplementary Files [file 41467_2023_36615_MOESM3_ESM.pdf]

Description of Additional Supplementary Files

File Name: Supplementary Data 1
Description: Alignment file for maximum likelihood tree of *Sox* HMG domains.

File Name: Supplementary Movie 1
Description: Discharge of large piercing cells (nematocytes) from the tentacle tips of a wild type polyp using an infrared laser ablation system. Note dust on the lens that appears in the still images in Figure 5d.

File Name: Supplementary Movie 2
Description: Discharge of large piercing cells (nematocytes) from the tentacle tips of an *NvSox2* mutant polyp using an infrared laser ablation system. Note dust on the lens that appears in the still images in Figure 5d.
