# Supplementary material for: Single-cell atavism reveals an ancient mechanism of cell type diversification in a sea anemone
Source: Nat Commun. 2023 Feb 16;14:885. doi: 10.1038/s41467-023-36615-9 (PMC9935875; doi:10.1038/s41467-023-36615-9)
Supplement: Supplementary file 7 — Reporting Summary [file 41467_2023_36615_MOESM7_ESM.pdf]

nature portfolio

Corresponding author(s): Leslie S Babonis

Last updated by author(s): 15 Dec 2022

Reporting Summary

Nature Portfolio wishes to improve the reproducibility of the work that we publish. This form provides structure for consistency and transparency in reporting. For further information on Nature Portfolio policies, see our Editorial Policies and the Editorial Policy Checklist.

Statistics

For all statistical analyses, confirm that the following items are present in the figure legend, table legend, main text, or Methods section.

n/a	Confirmed	
☐	☒	The exact sample size (*n*) for each experimental group/condition, given as a discrete number and unit of measurement
☐	☒	A statement on whether measurements were taken from distinct samples or whether the same sample was measured repeatedly
☐	☒	The statistical test(s) used AND whether they are one- or two-sided<br>*Only common tests should be described solely by name; describe more complex techniques in the Methods section.*
☒	☐	A description of all covariates tested
☒	☐	A description of any assumptions or corrections, such as tests of normality and adjustment for multiple comparisons
☐	☒	A full description of the statistical parameters including central tendency (e.g. means) or other basic estimates (e.g. regression coefficient) AND variation (e.g. standard deviation) or associated estimates of uncertainty (e.g. confidence intervals)
☐	☒	For null hypothesis testing, the test statistic (e.g. *F*, *t*, *r*) with confidence intervals, effect sizes, degrees of freedom and *P* value noted<br>*Give P values as exact values whenever suitable.*
☒	☐	For Bayesian analysis, information on the choice of priors and Markov chain Monte Carlo settings
☒	☐	For hierarchical and complex designs, identification of the appropriate level for tests and full reporting of outcomes
☒	☐	Estimates of effect sizes (e.g. Cohen's *d*, Pearson's *r*), indicating how they were calculated

Our web collection on statistics for biologists contains articles on many of the points above.

Software and code

Policy information about availability of computer code

Data collection	Imaris v 7.6.1 (Oxford Instruments, USA) was used for demarcating regions of interest for co-localization analyses.
Data analysis	Statistical analyses were performed using R (v 4.2.1) and Microsoft Excel (v 2 122). Fiji v 1.53f {ImageJ) was used for linear measurements of cell size and to adjust micrographs for brightness and contrast. Figures were prepared in FigTree v 1.4 and Adobe Illustrator v 24.1.1

For manuscripts utilizing custom algorithms or software that are central to the research but not yet described in published literature, software must be made available to editors and reviewers. We strongly encourage code deposition in a community repository (e.g. GitHub). See the Nature Portfolio guidelines for submitting code & software for further information.

Data

Policy information about availability of data

All manuscripts must include a data availability statement. This statement should provide the following information, where applicable:

- Accession codes, unique identifiers, or web links for publicly available datasets
- A description of any restrictions on data availability
- For clinical datasets or third party data, please ensure that the statement adheres to our policy

All data generated or analyzed during this study, including source data, are included in this published article (and its Supplementary Information files).

Human research participants

Policy information about studies involving human research participants and Sex and Gender in Research.

Reporting on sex and gender	*N/A*
Population characteristics	*N/A*
Recruitment	*N/A*
Ethics oversight	*N/A*

Note that full information on the approval of the study protocol must also be provided in the manuscript.

Field-specific reporting

Please select the one below that is the best fit for your research. If you are not sure, read the appropriate sections before making your selection.

☒ Life sciences ☐ Behavioural & social sciences ☐ Ecological, evolutionary & environmental sciences

For a reference copy of the document with all sections, see nature.com/documents/nr-reporting-summary-flat.pdf

Life sciences study design

All studies must disclose on these points even when the disclosure is negative.

Sample size	Cell and tissue level analyses were replicated at the individual level, numbers of replicate individuals for each assay are indicated in the figure captions. Two previous publications suggest the level of replication used here is sufficient to identify reproducible effects when present (Babonis et al. 2022 doi.org/10.1073/pnas.211370111, Babonis et al2019 doi.org/10.1186/s13227-017-0077-7)
Data exclusions	No data were excluded from this study
Replication	Electron micrographs are representative images of at least three cells of the indicated type sampled from at least two individuals. Exact replicates are provided in the Statistics and reproducibility section of the Methods. For fluroescent and light micrographs, replicates are indicated in the figure legends and represent the number of indivduals imaged for each condition or developmental stage. All attempts at replication were successful.
Randomization	There were no interventions associated with this study so randomization is not relevant
Blinding	There were no interventions so blinding is not relevant

Reporting for specific materials, systems and methods

We require information from authors about some types of materials, experimental systems and methods used in many studies. Here, indicate whether each material, system or method listed is relevant to your study. If you are not sure if a list item applies to your research, read the appropriate section before selecting a response.

Materials & experimental systems

n/a	Involved in the study
☐	☒ Antibodies
☒	☐ Eukaryotic cell lines
☒	☐ Palaeontology and archaeology
☐	☒ Animals and other organisms
☒	☐ Clinical data
☒	☐ Dual use research of concern

Methods

n/a	Involved in the study
☒	☐ ChIP-seq
☒	☐ Flow cytometry
☒	☐ MRI-based neuroimaging

Antibodies

Antibodies used	anti-minicollagen4, a kind gift from the lab of Suat Ozbek (Heidelberg)
Validation	Zenkert, C., Takahashi, T., Diesner, M.-O. & Özbek, S. Morphological and molecular analysis of the Nematostella vectensis cnidom. PLoS ONE 6, e22725 (2011).

Animals and other research organisms

Policy information about studies involving animals; ARRIVE guidelines recommended for reporting animal research, and Sex and Gender in Research

Laboratory animals	starlet sea anemone Nematostella vectensis, ages: zygote to 2yr old adult
Wild animals	This study did not involve wild-caught animals
Reporting on sex	all analyses were performed in randomly selected juveniles before they reached sexual maturity. it is not possible to determine the sex of these animals before they mature.
Field-collected samples	no samples were collected from the field
Ethics oversight	no ethical approval was required because these are unregulated marine invertebrates

Note that full information on the approval of the study protocol must also be provided in the manuscript.
